# Hormonal mechanisms of women’s risk in the face of traumatic stress

**DOI:** 10.1073/pnas.2524903122

**Published:** 2025-12-15

**Authors:** Jennifer S. Stevens, Madeline Davis, Cecilia A. Hinojosa, Rebecca Hinrichs, Alyssa R. Roeckner, Katelyn I. Oliver, Linzie Taylor, Justin L. C. Santos, Helena Zeleke, Esther Lin, Kristina Dahlgren, Timothy D. Ely, Amy R. Murphy, Colin Johnson, Dasani DelRosario, Natalie Merrill, Xing Zhang, Kelly F. Ethun, Marisa Young, Andrea Braden, Nicole R. Nugent, Abigail Powers, Sanne J. H. van Rooij, Kim Wallen, Vasiliki Michopoulos

**Affiliations:** ^a^Department of Psychiatry and Behavioral Sciences, Emory University School of Medicine, Atlanta, GA 30329; ^b^Center for Visual and Neurocognitive Rehabilitation, Joseph Maxwell Cleland Atlanta Veterans Affairs Medical Center, Atlanta, GA 30033; ^c^Department of Psychology, University of New Mexico, Albuquerque, NM 87106; ^d^Biomarkers Core, Emory National Primate Research Center, Emory University, Atlanta, GA 30329; ^e^Department of Pathology and Laboratory Medicine, Emory University School of Medicine, Atlanta, GA 30322; ^f^Department of Gynecology and Obstetrics, Emory University School of Medicine, Atlanta, GA 30322; ^g^Obstetrics and Gynecology Hospitalist Services, TeamHealth, Knoxville, TN 37916; ^h^Department of Psychiatry and Human Behavior, Brown University, Providence, RI 02903; ^i^Department of Psychology, Emory College, Atlanta, GA 30322

**Keywords:** posttraumatic stress disorder, fMRI, women’s health, anxiety, hormones

## Abstract

Women are underrepresented in biomedical research, limiting understanding of their disproportionate rates of stress-related disorders. Although men experience more trauma, women are twice as likely to develop posttraumatic stress disorder (PTSD). We tested whether ovarian hormones contribute to this disparity via their influence on threat-related brain activity. In a randomized, double-blind crossover study of 110 young women, we examined how exogenous estradiol (E2) modulates threat neurocircuitry, timed precisely to ovarian phase. E2 increased engagement of the ventromedial prefrontal cortex and, in the early luteal phase, reduced central and corticomedial amygdala reactivity to threat in women with little trauma history, but not those with PTSD. Fluctuations in E2 therefore modulate neural responses to threat, and sensitivity to these fluctuations may underlie women’s heightened vulnerability to stress-related disorders.

## Significance

Estradiol, the primary estrogen produced by the ovaries, has long been thought to influence emotional functioning in women. We predicted that cyclical fluctuations in estradiol might exacerbate neural responsivity to threat among women with a history of trauma, explaining women’s increased risk for posttraumatic stress disorder (PTSD). We tested this by administering transdermal estradiol to examine its effect on affective brain regions such as the amygdala. When estradiol was naturally low, women with PTSD showed hyperreactivity of the central amygdala. However, estradiol administration caused a reduction in central and corticomedial amygdala reactivity only among women with little experience of trauma, with no effect among women with PTSD. Chronic stress may therefore reduce estradiol’s beneficial effects on amygdala function.

Women experience stress-linked disorders at up to twice the rate of men, yet the biological mechanisms that contribute to this disparity remain poorly understood (1–4). Despite decades of research on stress and mental health, basic and biomedical studies have historically focused on males, leaving a critical gap in our knowledge of how cycling ovarian hormones may influence vulnerability to chronic mental health conditions (5–7). Estradiol (E2), in particular, can dampen physiological emotional arousal (8) and change the ways that emotional memories are encoded and regulated (9). This is highly relevant to posttraumatic stress disorder (PTSD) which is characterized by heightened arousal, intrusive emotional memory, and hyperreactivity of threat-related neurocircuitry (10–13). However, little research has provided a causal test of the effects of E2 on neural responses to threat cues in humans, particularly those with PTSD. Here, we conducted a randomized placebo-controlled within-subject cross-over study using acute exogenous E2 administration to examine its modulation of threat-related neural responses in women.

Preclinical evidence suggests that E2 can influence stress and physiological arousal responses to threat cues. At a moderate dose or during the endogenous preovulatory peak, E2 has reliable stress- or arousal-dampening effects that are mediated by the β-estrogen receptor (ERβ) (14–16). But at higher doses, E2 can increase stress or arousal unrelated to ERβ (15), driven instead by a disruption of normal affective inhibition via the ERα (17). The E2-anxiety association therefore follows an inverted U-shaped curve. In primates, estrogen receptors populate brain regions involved in regulating threat responses, with the amygdala (particularly the centromedial nuclei) predominantly populated by ERα (18–21) and the ventromedial prefrontal cortex (vmPFC) and hippocampus preferentially populated by ERβ (18, 21). E2 is required for stress-related dendritic remodeling of vmPFC-basolateral amygdala projections in female rodents (22), supporting its role in regulation of affective responses. Concordantly, in healthy reproductive-aged women, fluctuations in endogenous E2 are associated with changes in the neural processing of threat cues, with reduced amygdala reactivity to threat in higher-E2 phases of the ovarian cycle (23–25). Together, findings from observational studies to date suggest that conditions of higher E2 facilitate down-regulation of amygdala reactivity [with an exception for long-term suppression of E2 under oral contraceptives; e.g., (26)]. However, among healthy young women studied in the early follicular phase when E2 is naturally low, exogenous E2 had no effect on amygdala reactivity to negative affective stimuli (27, 28).

It is as yet unclear whether trauma and posttraumatic stress potentiate the effects of E2 on threat neurocircuitry. Amygdala reactivity to threat cues is a risk factor for stress vulnerability (29–32) that can predate trauma exposure and predict future PTSD and internalizing (11, 13, 33, 34). One determinant of amygdala hyperreactivity may be sensitivity of this region to changes in E2, or changes in the inhibitory input to the amygdala from the vmPFC. Several observational studies suggest that PTSD-related hyperarousal is most apparent among women with low endogenous E2, or during lower-E2 phases of the ovarian cycle including the early follicular and early luteal phases (35, 36). No study has examined the effects of exogenous E2 on amygdala reactivity among trauma-exposed participants.

## Results

### Study Design.

**Fig. 1.**
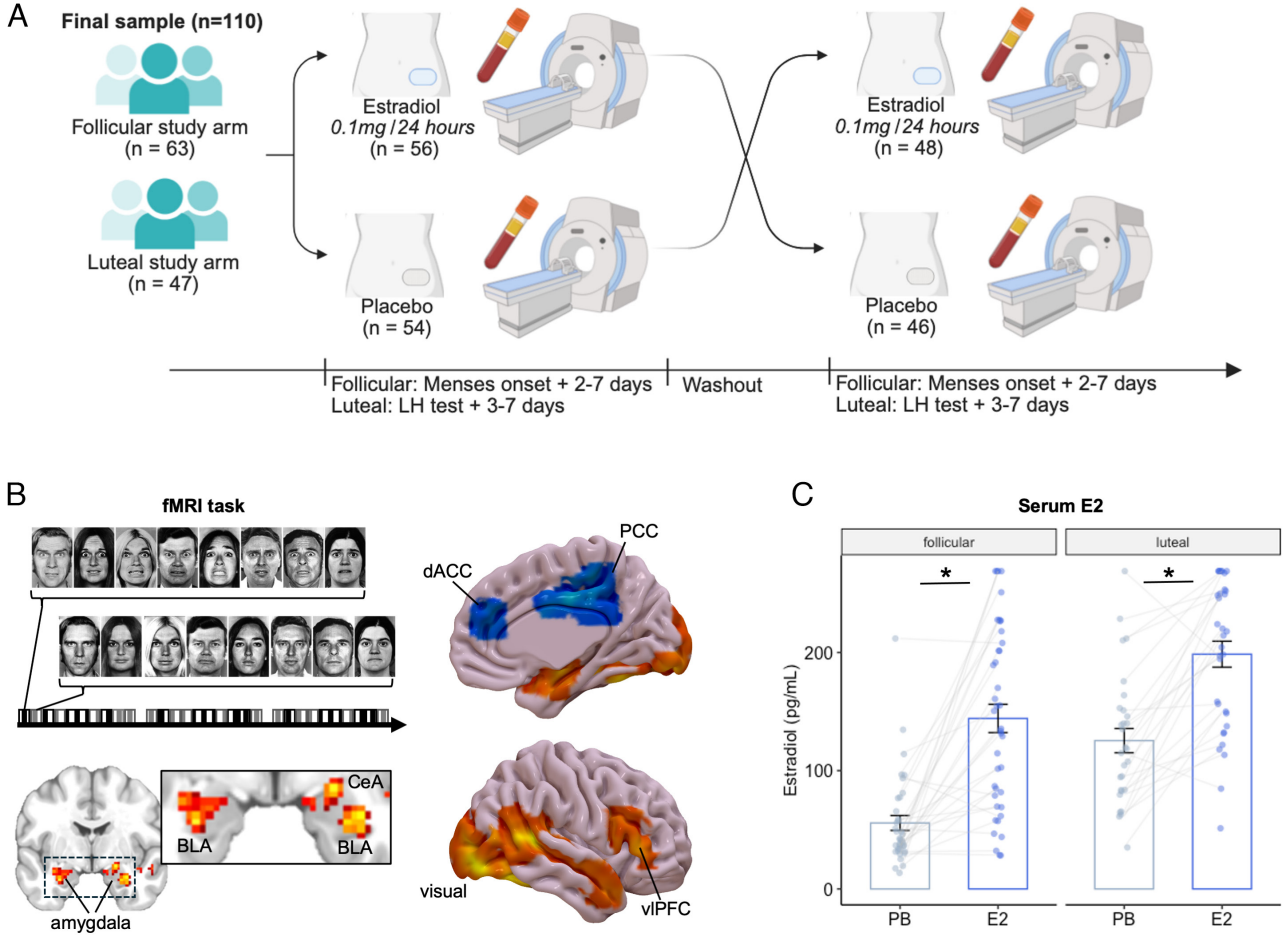
Study design. (*A*) Timing of patch administration and study procedures. (*B*) FMRI task, including whole-brain responses across full set of 203 scan visits, at cluster-corrected *p*_FWE_ < 0.05. Fearful faces, relative to neutral faces, were associated with greater activation of bilateral amygdala (*Left* Z = 5.16, xyz = −32, −10, −16, k = 131; *Right* Z = 4.55, xyz = 24, −10, −16, k = 187), bilateral visual cortex (*Left* Z = 6.44, xyz = −42, −60, −16, k = 2,651; *Right* Z = 5.75, xyz = 46, −42, 9, k = 637), and *Right* ventrolateral prefrontal cortex (vlPFC; Z = 4.76, xyz = 48, 18, 19, k = 269), and lesser activation of posterior cingulate cortex (PCC; Z = 4.55, xyz = 1, −18, 36, k = 374) and dorsal anterior cingulate cortex (dACC; Z = 3.91, xyz = 11, 38, 16, k = 142). The amygdala clusters overlapped both BLA and CeA. Color scale: Red-yellow shows t-score range 2 to 5; blue-green shows range −2 to −5. (*C*) The E2 patch produced an increase in serum estradiol relative to placebo, with similar levels of increase observed in both the early follicular phase and the early luteal phase [E2 patch *M*(*SD*) = 176.06(89.28) pg/mL; PB patch: 88.66(63.58) pg/mL; *t* = 6.52, *P* < 0.001]. There was no interaction of patch condition with cycle phase, *P* = 0.51. Serum estradiol levels were higher in the cohort scanned in the early luteal vs. the early follicular phase of the cycle [*t* = 4.19, *P* < 0.001; luteal: *M*(*SD*) = 171.59(84.96) pg/mL; follicular: 105.82(82.31) pg/mL].

Here, we conducted a double-blind randomized controlled within-subject trial of a single high-dose administration of E2, to examine its acute effects on neural responses to social threat cues in women, and on the PTSD-related pattern of heightened amygdala and reduced vmPFC reactivity to threat (Fig. 1; clinicaltrials.gov ID: NCT03973229) (37). Reproductive-aged women not taking any hormonal or psychiatric medications (*N* = 110 with 203 scan visits; Table 1, cohort diagram *SI Appendix*, Fig. S1) were randomized to receive either E2 or placebo during a phase of the ovarian cycle when endogenous E2 is typically low (early follicular phase during menses or early luteal phase shortly after ovulation). They crossed over to the other study condition (E2 or placebo) during the same time of the ovarian cycle on the following month. Participants were Black and/or African American women, who often experience disproportionately higher rates of trauma and other major stressors (38), and as a result, bear a greater burden of PTSD.

**Table 1.** Demographic and clinical characteristics of the sample, *n* = 110

Characteristic	TC (n = 40)	NLT (n = 36)	PTSD+ (n = 34)	*P*
Cycle phase, n				
Early Luteal	16	17	14	0.798
Early Follicular	24	19	20	
Age, M (SD)	26.25 (5.30)	25.11 (4.60)	26.06 (4.86)	0.572
BMI, M (SD)	27.30 (6.00)	25.14 (4.42)	26.47 (5.88)	0.232
Age at menarche, M (SD)	11.75 (1.61)	11.81 (1.33)	11.85 (1.46)	0.956
Employment status, n (%)				0.731
Unemployed	12 (30.0)	13 (36.1)	12 (35.3)	
Disabled	2 (5.0)	0 (0.0)	1 (2.9)	
Employed or student	26 (65.0)	23 (63.9)	21 (61.8)	
Relationship status, n (%)				0.942
Single, never married	35 (87.5)	34 (94.4)	31 (91.2)	
Married	2 (5.0)	1 (2.8)	1 (2.9)	
Divorced	2 (5.0)	1 (2.8)	1 (2.9)	
Domestic partner	1 (2.5)	0 (0.0)	1 (2.9)	
Education, n (%)				0.891
<12th grade	2 (5.0)	1 (2.8)	1 (2.9)	
High school graduate or equivalent	9 (22.5)	8 (22.6)	9 (26.5)	
Some college or technical school	9 (22.5)	10 (27.8)	9 (26.5)	
Technical school graduate	4 (10.0)	1 (2.8)	1 (2.9)	
College graduate	10 (25.0)	11 (30.6)	12 (35.3)	
Graduate school	6 (15.0)	5 (13.9)	2 (5.9)	
Income, n (%)				0.007
$0 to 499	3 (7.5)	7 (19.4)	8 (23.5)	
$500 to 999	4 (10.0)	3 (8.3)	2 (5.9)	
$1,000 to 1,999	7 (17.5)	4 (11.1)	7 (20.6)	
>=$2000	24 (60.0)	13 (36.1)	13 (38.2)	
Missing	2 (5.0)	9 (25.0)	4 (11.8)	
Has health insurance, n (%)				0.326
No	7 (17.5)	6 (16.7)	5 (14.7)	
Yes—Medicaid	12 (30.0)	5 (13.9)	8 (23.5)	
Yes—Private	19 (47.5)	24 (66.7)	17 (50.0)	
Yes—Both	1 (2.5)	1 (2.8)	4 (11.8)	
Missing	1 (2.5)	0 (0.0)	0 (0.0)	
Handedness, n (%)				0.051
Left or ambidextrous	6 (15.0)	4 (11.1)	2 (5.9)	
Right	34 (85.0)	32 (88.9)	32 (94.1)	
# types of potentially traumatizing events, M (SD)	4.30 (1.64)	1.70 (1.42)	5.24 (2.35)	<0.001
Childhood maltreatment, M (SD)	46.00 (13.22)	34.83 (9.46)	63.62 (18.12)	<0.001
Dissociative symptom severity, M (SD)	0.05 (0.32)	0.00 (0.00)	0.50 (0.75)	<0.001
Trait anxiety, M (SD)	36.92 (11.08)	38.41 (10.28)	47.76 (11.11)	<0.001
Lifetime (not current) PTSD, n (%)	12 (30.0)	0 (0.0)	0 (0.0)	0.002
Current MDE, n (%)	2 (5.0)	3 (8.3)	18 (52.9)	<0.001
Current AUD, n (%)	2 (5.0)	1 (2.8)	3 (8.8)	0.531
Current SUD (%)	5 (12.5)	1 (2.8)	4 (11.8)	0.274
Proportion volumes >1 mm FD in fMRI task, M (SD)	0.007 (0.017)	0.004 (0.010)	0.002 (0.005)	0.188

Abbreviations: M = mean, SD = standard deviation, TC = trauma-exposed control group, NLT = no- and low-trauma control group, PTSD+ = PTSD and subthreshold group, CTQ = Childhood Trauma Questionnaire, MDE = major depressive episode, AUD = alcohol use disorder, SUD = substance use disorder, FD = framewise displacement, fMRI = functional MRI.

### Effects of Exogenous E2 Administration On Circulating Hormone Levels.

We confirmed that the E2 patch produced an increase in serum estradiol (Fig. 1*C*), relative to placebo (PB). Serum estradiol was higher in the cohort scanned in the early luteal vs. the early follicular phase of the ovarian cycle. There was no interaction of patch condition with cycle phase. E2 administration had no effect on progesterone (P4) or testosterone but did produce an increase in estrone (*SI Appendix*, Fig. S2). As expected, P4 was higher in the early luteal phase than in the early follicular phase, and this was also the case for testosterone.

### Effects of Exogenous E2 Administration On Neural Processing of Social Threat Cues.

**Fig. 2.**
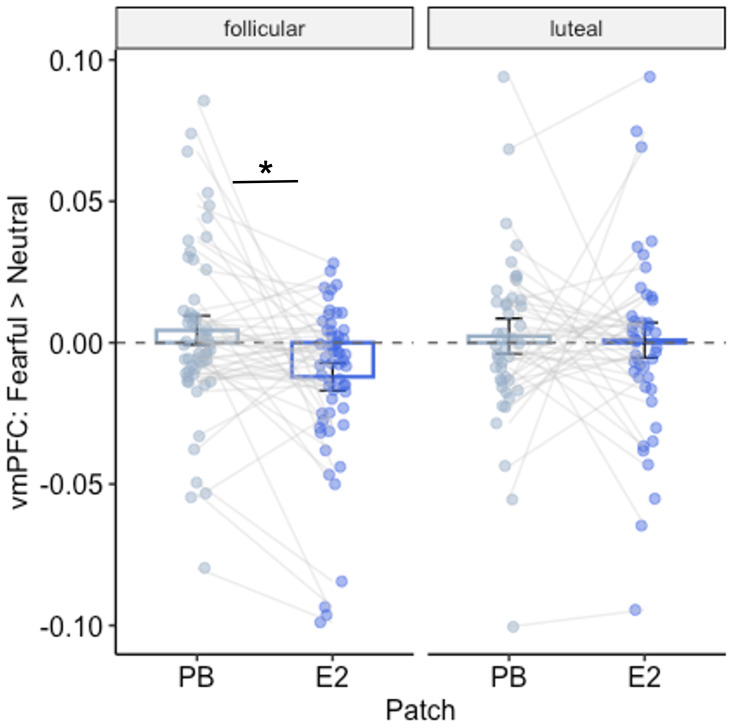
Effect of exogenous E2 on the vmPFC response. The E2 patch produced a preferential response to neutral vs. fearful faces in the vmPFC (E2 main effect *t* = −3.17, *P* = 0.002, d = −0.60; E2 × phase *P* = 0.06). On the y-axis, negative values indicate greater vmPFC response to neutral vs. fearful faces. Disaggregated analyses showed that the E2 effect was specific to the early follicular phase of the cycle (*t* = −3.48, *P* < 0.001, d = −0.66). E2 had no effect on the vmPFC response in the early luteal phase (*P* = 0.83).

We then examined how E2 modulated neural processing of social threat cues in the early follicular and early luteal phases of the ovarian cycle, in the full sample (*n* = 110). The primary outcome of interest was the fMRI BOLD response to fearful > neutral face stimuli, extracted from volume-averaged basolateral amygdala (BLA), central amygdala (CeA), corticomedial amygdala (ComA), and vmPFC regions of interest (ROIs), with *p*-values < 0.007 considered significant after Bonferroni correcting for 7 ROIs. E2 administration produced a stronger vmPFC response to neutral versus fearful faces, which was not observed under PB (Fig. 2 and *SI Appendix*, Table S1). Planned disaggregated analyses of each ovarian phase showed that this effect was prominent in the early follicular phase, but not in the early luteal phase. There were no main effects of E2 administration or interactions with cycle phase in the BLA, ComA, or CeA.

### Trauma- and PTSD-Related Differences in Women’s Neural Processing of Social Threat Cues.

**Fig. 3.**
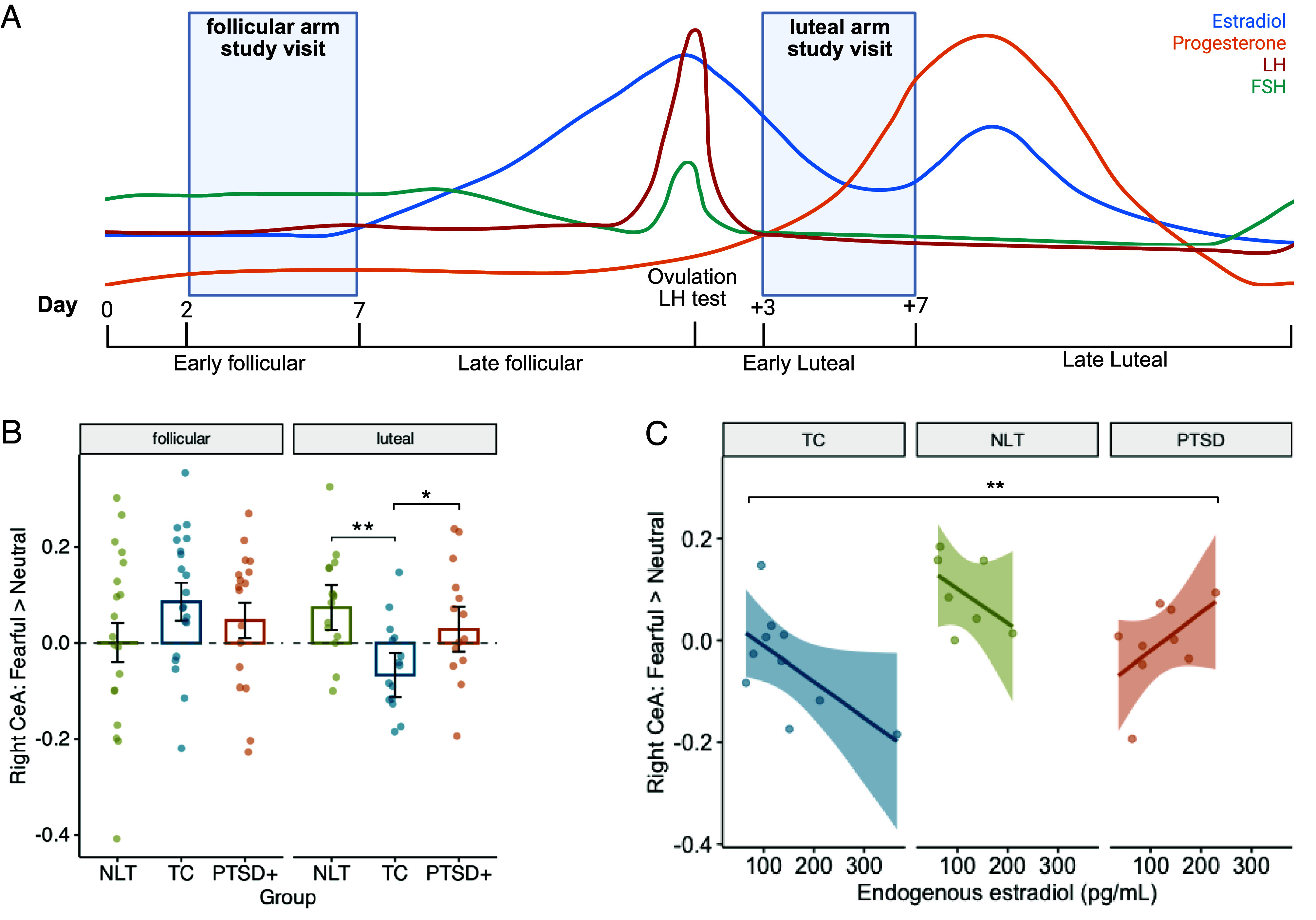
Group differences in the response to social threat cues, under endogenous hormonal conditions (placebo). (*A*) Schematic diagram of study visits relative to prototypical hormonal fluctuation over the ovarian cycle. *B.* The three groups all showed right CeA reactivity to social threat cues (fearful > neutral faces) which were higher in the early follicular phase (follicular vs. luteal phase *t* = 3.16, *P* = 0.002, group effect in follicular phase *P* = 0.30). However, in the early luteal phase, the PTSD+ and NLT groups showed greater right CeA threat reactivity than TC (NLT vs. TC *t* = 3.71, *P* = 0.0006, PTSD+ vs. TC: *t* = 2.48, *P* = 0.02). (*C*) In the luteal phase, endogenous estradiol was negatively related to right CeA reactivity in the TC and NTC groups, but positively related in PTSD+ (endogenous E2 × group *t* = 2.26, *P* = 0.04; overall model R^2^Δ = 0.55). TC = Trauma-exposed control, NLT = No- and low-trauma control, PTSD = PTSD and subthreshold.

We then tested for the PTSD-linked pattern of amygdala hyperreactivity. We predicted that women with PTSD would show hyperreactivity in the early follicular phase with consistently low endogenous E2, as well as in the early luteal phase when E2 rapidly drops after ovulation (Fig. 3*A*). A group with PTSD and subthreshold PTSD (PTSD+) were compared to a control group with comparable trauma exposure history (trauma-exposed control; TC), and a control group with little to no trauma exposure (NLT; groups further described in Table 1 and *Materials and Methods*). Group differences were investigated within the placebo scan condition, which was considered a “baseline” for this purpose.

Among the primary ROIs, the PTSD+ and NLT groups showed greater threat reactivity in the right CeA compared to TC, but only within the early luteal phase (Fig. 3*B*). In the follicular phase, all three groups showed relatively high right CeA responses to social threat cues, which did not differ by group. A similar pattern was observed for the right ComA, but the effect did not survive Bonferroni correction (NLT group × cycle phase, *t* = 2.73, *p*_uncorr_ = 0.008; PTSD group × cycle phase, *t* = 1.41, *p*_uncorr_ = 0.16). There were no group differences in the BLA, vmPFC, left ComA, or left CeA.

To test whether group differences in the early luteal phase were related to either the higher level of endogenous E2, or the rise in endogenous P4, we then tested for linear associations between endogenous hormones and the magnitude of right CeA reactivity to threat. While there was no association with P4 (P4 *P* = 0.97; P4 × group *P* = 0.63), there was an endogenous E2 × PTSD+ group effect (Fig. 3*C*). Endogenous E2 was negatively related to early luteal CeA reactivity in TC and NLT groups but positively related to CeA reactivity in the PTSD+ group. This suggested that women with PTSD may show less E2 regulation of the right CeA response to threat.

### Effects of Exogenous E2 on Neural Response to Social Threat Cues Across the PTSD, Trauma-Exposed, and Low-trauma Groups.

**Fig. 4.**
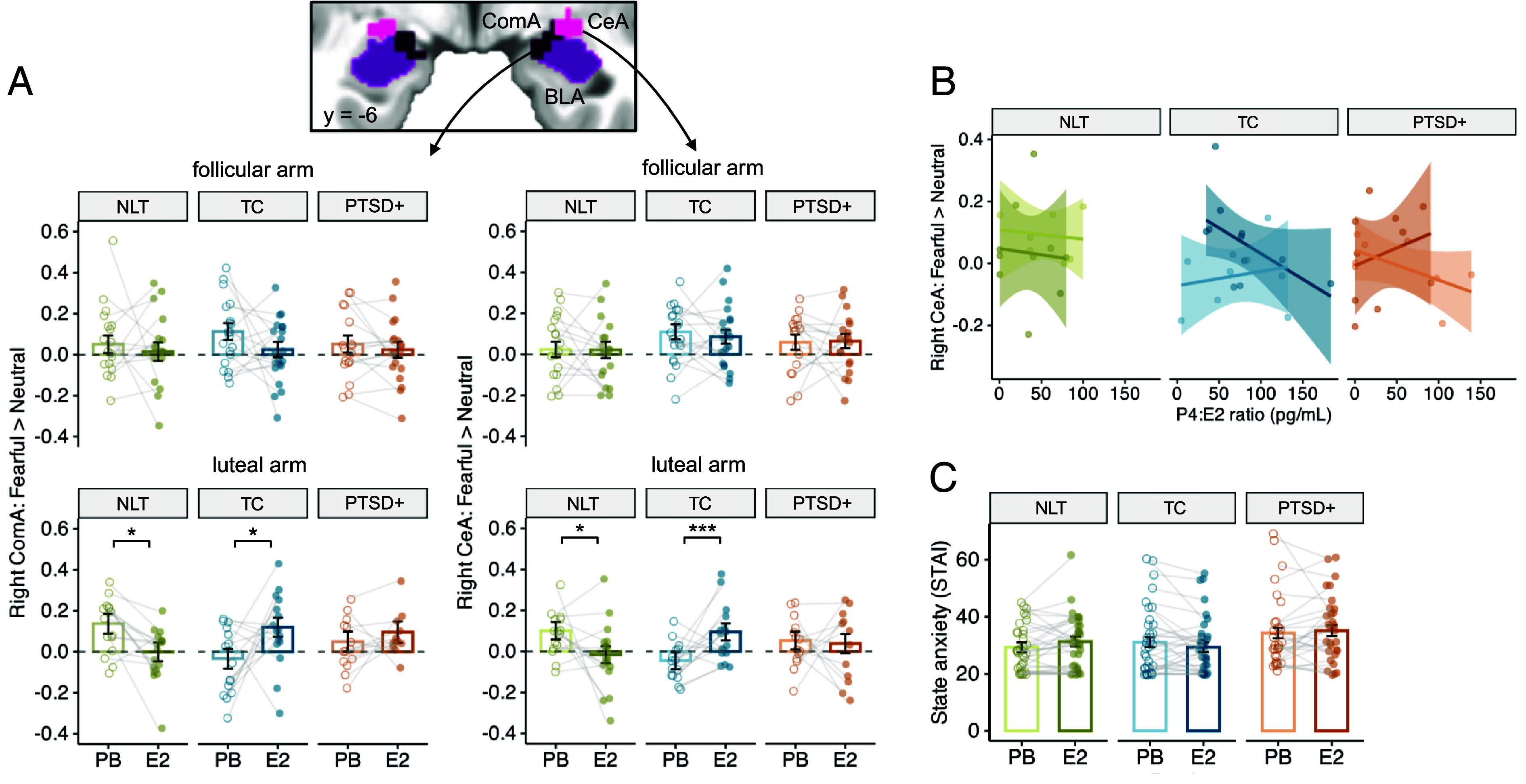
Group-specific effects of E2 administration. (*A*) Effects of exogenous E2 administration on the right amygdala responses to fearful>neutral faces, separated by ovarian cycle phase. There was an E2 patch × phase × group interaction (*t* = −2.86, *P* = 0.005) in right CeA. In the early follicular phase, right CeA responses were not influenced by E2 patch (E2 *P* = 0.68; E2 × group *P* = 0.79). In the early luteal phase, exogenous E2 dampened the right CeA response in the NLT group (E2 × NLT group *t* = −4.01, *P* < 0.001; E2 effect in NLT: *b*(*SE*) = −0.12(0.05), *P* = 0.03, d = −0.85), and magnified the response in the TC group (E2 effect in TC: *b*(*SE*) = 0.16(0.03), *P* = 0.0002, d = 1.90). In the PTSD+ group, E2 had a different effect than in TC (E2 × PTSD+ group *t* = −2.27, *P* = 0.02), such that there was no E2 effect in PTSD+: −0.02(0.05), *P* = 0.79, d = −0.10). Similar effects were observed for the right ComA (NLT: *b*(*SE*) = −0.14(0.06), *P* = 0.02, d = −0.84, TC: *b*(*SE*) = 0.15(0.06), *P* = 0.02, d = 0.93, PTSD+: *b*(*SE*) = 0.05(0.06), *P* = 0.48, d = 0.28). *B.* In the luteal phase, lower P4/E2 ratio in serum was linked with higher right CeA reactivity in the TC group, whereas it was linked with lower right CeA reactivity in the PTSD+ group (E2 patch × P4/E2 ratio × PTSD+ group *t* = 2.11, *P* = 0.04). There was no association of either P4 or the P4/E2 ratio with right ComA reactivity. (*C*) Group moderated the effects of E2 administration on state anxiety (E2 patch × PTSD+ group *t* = 2.43, *p* = 0.02), but post hoc tests showed no significant effects of E2 in any group (*p*s > 0.05).

E2 administration differentially influenced threat reactivity of the right CeA and right ComA in the PTSD+, TC, and NLT groups (Fig. 4*A* and *SI Appendix*, Table S2). In the early follicular phase, E2 administration had no effect on right CeA or right ComA reactivity in any group. However, in the early luteal phase, E2 administration produced a decrease in right CeA and ComA reactivity in the NLT group, relative to TC. The TC group showed low right CeA and ComA reactivity under placebo, with an unexpected increase in reactivity under E2. The PTSD+ group showed no increase or decrease under E2.

Follow-up tests were then conducted to test whether group-dependent effects of E2 administration in the luteal phase were related to circulating hormone levels of either E2, P4, or the P4/E2 ratio. There was no association of the right CeA or ComA response with circulating P4 or E2 levels, but there was an association with the P4/E2 ratio for the CeA only. The effect of the E2 patch was moderated by the resulting P4/E2 ratio in the PTSD+ group (Fig. 4*B*).

In the right BLA, E2 patch effects were also moderated by group and cycle phase (E2 × group × cycle phase *t* = −2.26, *P* = 0.05). However, this did not meet the Bonferroni-corrected threshold, and we did not examine the effect further. There were no significant E2 patch × group effects on the left CeA, left ComA, left BLA, or vmPFC. Exploratory whole-brain analysis of regions outside the ROIs was conducted for the primary test of E2 × group × cycle phase, but there were no additional regions that met the corrected threshold.

Importantly, participants’ trauma exposure history or current PTSD did not influence the resulting circulating estradiol concentration following patch administration (E2 patch effect on serum estradiol in PTSD+ vs. TC group *P* = 0.48, E2 patch effect in NTC vs. TC group *P* = 0.11).

In summary, the right CeA and right ComA showed trauma- and PTSD-linked effects of E2 administration. In these amygdala nuclei, reactivity to threat cues was higher in the early follicular vs. early luteal phase but was not influenced by exogenous E2 administration in the follicular phase. In the early luteal phase, E2 administration dampened right CeA and ComA reactivity only in the NLT group and produced a surprising increase in reactivity in the TC group. When exogenous E2 pushed the P4:E2 ratio lower, it appeared to increase right CeA reactivity in TC.

### Effects of E2 on Self-Reported Symptoms of Anxiety and PTSD.

We predicted that E2 might also influence self-reported anxiety. A state-specific effect was expected given that participants only wore the patch for 24 to 48 h prior to the scan. E2 administration did have differential effects on self-reported state anxiety across the 3 groups (Fig. 4*C*) but post hoc tests showed no E2 effects in any group. Moreover, there was no effect of E2 administration on the more stable metrics of trait anxiety (E2 patch × PTSD group *P* = 0.97), or PTSD symptom severity over the past 30 d (E2 patch × PTSD group *P* = 0.71). Effects on depression are reported in *SI Appendix*, Supplementary Text (not significant).

## Discussion

This comprehensive study experimentally manipulated E2 levels, demonstrating effects of E2 on the neural response to social threat cues in women, as well as the potential for E2 to influence threat processing differentially among women exposed to trauma and those with PTSD. Strengths of the study include the rigorous experimental double-blind case–control design, focus on two ovarian cycle phases when E2 is naturally low (early follicular phase, early luteal phase), and examination of E2’s modulation of neural threat reactivity following trauma and PTSD.

When examining the effects of E2 among all participants irrespective of trauma history or symptoms, exogenous E2 promoted preferential vmPFC engagement to neutral social cues. The vmPFC’s engagement for neutral cues has parallels in other paradigms, particularly those involving safety learning. For example, the vmPFC is more responsive to learned safety cues (CS−) versus learned threat (CS+) (39) and is critical for context-dependent fear extinction (40). One of the few prior studies to examine the effects of exogenous E2 on brain function in reproductive-aged women showed an enhancing effect on vmPFC engagement during fear extinction recall (41), which involves the formation and stabilization of a safety memory. Acute E2 administration therefore may promote a role for the vmPFC in regulating emotional responses during the presentation of safety-like stimuli. E2 effects on vmPFC function may be mediated by binding to ERβ (18, 42) due to its proliferation within this cortical area or by fast effects on membrane-bound receptors such as GPR30 (43).

In addition, findings from the placebo condition provide new evidence about phases of the ovarian cycle in which women with trauma and PTSD might show amygdala hyperreactivity to threat cues. In the current study, the timing of the MRI visits was precisely yoked to the cycle using daily prospective monitoring and luteinizing hormone (LH) tests to confirm the timing of ovulation. Prior studies in which there was no control of cycle timing, hormonal medication use, or reproductive age showed threat hyperreactivity of the right amygdala in women with PTSD relative to trauma-exposed control participants (10, 44), with overlap of the CeA and ComA. The current study isolated PTSD-related amygdala hyperreactivity to the early luteal phase, particularly within right CeA. This was primarily driven by the fact that the TC group showed lower CeA reactivity than other groups in the early luteal phase, corresponding with a negative linear association with endogenous E2.

Finally, the findings addressing differential neural effects of exogenous E2 on trauma-exposed women and those with PTSD provide a causal test of the role of cyclical fluctuations in women’s risk for stress-related disorders. Current theoretical models suggest that low E2, or lower neural sensitivity to E2, may promote PTSD symptoms by either increasing threat reactivity, or inhibiting contextual learning processes such as fear extinction (3, 4). Here, we tested the first component of this model, focusing on acute effects on threat reactivity. Exogenous E2 appeared to show trauma-dependent effects on the right CeA and ComA, such that the E2 patch did not produce a decrease in threat reactivity in the TC and PTSD+ groups. Chronic stress following trauma and PTSD diagnosis may alter the function of ERs in the CeA and ComA, or change inputs to these nuclei from ER-rich regions such as the bed nucleus of the stria terminalis (45, 46). Stress can regulate ER transcription via glucocorticoid-mediated transcriptional repression (47, 48). Given the proliferation of ERα in these amygdala nuclei (18,19, 20, 49), future translational work should focus on the effects of stress in modulating the sensitivity of ERα and potentiating threat responses. Estrogen treatment can reciprocally increase expression of stress-relevant receptors such as corticotropin-releasing hormone (*Crh*) and pituitary adenylate cyclase-activating polypeptide type 1 (*PAC1*) (50, 51), and induce corticosterone release when binding ERα (52), which may produce larger BOLD responses in fMRI.

Several findings suggested that high E2 may in fact potentiate threat reactivity following repeated trauma or PTSD. The PTSD+ group showed elevated state anxiety following E2 administration, as well as a positive relationship between endogenous E2 and right CeA reactivity in the placebo condition. In addition, while the NLT group showed a dampening of right CeA/ComA reactivity under E2 in the luteal phase, the TC group showed the opposite pattern, with an increase in right CeA/ComA reactivity under E2. Together, these findings suggest that trauma may produce greater E2 sensitivity, pushing women toward the right hand of the u-shaped curve where high levels of E2 promote rather than reduce anxiety-like responses. This stands in contrast to prior work showing that PTSD symptoms are correlated with greater physiological arousal only among women with lower endogenous E2 (35). The discrepancy may result because women in the prior study spanned reproductive and perimenopausal years, some reporting irregular menstrual cycles, so the cycle phase was not constrained.

The current findings must be interpreted in the context of a few notable limitations. First, this work was conducted in a sample of Black or African American women, who represent a group disproportionately affected by trauma and PTSD. Although we anticipate that the findings will generalize to other populations, this should be tested in future work. Finally, the PTSD+ group also included some women with subthreshold diagnoses, who experience high levels of distress and impairment (53), but may not be fully representative of the most severe cases of PTSD.

Together, the findings provide experimental evidence to suggest that E2 can modulate reactivity of the right CeA to increase PTSD risk among women. This, along with the more general finding showing that E2 promotes safety-like responding of the vmPFC in the context of social threat, advances our understanding of how E2 influences negative affect in women.

## Materials and Methods

### Participants.

*N* = 250 women ages 18 to 35 who reported Black or African American racial identity were enrolled from November 2019 to August 2024, with data collection concluding in January 2025. Participants were recruited through the Grady Trauma Project (GTP), a large longstanding community-based study of civilian trauma and PTSD conducted at Grady Memorial Hospital in Atlanta, GA, as well as local advertisements. All participants reported regular menstrual periods and were not taking any form of hormonal medication. All participants gave written consent following an informed consent discussion prior to any study procedures, and study protocols were approved by the Emory University Institutional Review Board and the Grady Research Oversight Committee. All procedures complied with the ethical standards of the Helsinki Declaration of 1975, as revised in 2008.

#### Inclusion criteria.

Black and African American women who had experienced a menstrual period within the past 60 d. Women had a smartphone and were willing to track menstrual cycles daily

#### Exclusion criteria.

Any form of hormone-based birth control or other hormonal medication, pregnancy, or breastfeeding. Current psychoactive medication use, current nicotine use or smoking, hypercoagulable conditions, history of embolism, current symptoms of psychosis or bipolar disorder, history of major head injury or neurological disorder, weight > 250 lb (to allow participants to fit comfortably inside the bore of the MRI machine), and typical physical contraindications for MRI such as metal implants.

#### Final sample.

As shown in the CONSORT diagram (*SI Appendix*, Fig. S1), *n* = 126 women were randomized to receive either an estradiol or placebo patch. *N* = 16 were randomized and received a patch but dropped out prior to completing a neuroimaging visit (reasons for dropout in *SI Appendix*, Fig. S1). Characteristics of the final sample of *n* = 110, representing 203 scan visits, are further described in Table 1.

#### Group definition.

The PTSD+ group (n = 34) included participants diagnosed with current PTSD (n = 20) and subthreshold PTSD (n = 14) based on the Clinician Administered PTSD Scale for DSM-5 (54). Subthreshold PTSD included those who met symptom criteria in 3 of 4 symptom categories (among DSM-5 BCDE criteria), as well as criterion A (trauma exposure), F (duration), and G (functional impairment), following established methods (53). The trauma-exposed control group (TC, n = 40) included healthy participants who had a history of DSM-5 Criterion A trauma exposure but did not meet criteria for current PTSD. The no- and low-acuity-trauma group (NLT, n = 36) included individuals who were free of any psychiatric diagnosis and endorsed either no trauma exposure or a single lower-acuity traumatic experience, such as a single car accident without injuries. Psychological assessments are further outlined in *SI Appendix*, *Supplemental Methods*.

### Procedure.

This was a double-blind, placebo-controlled, within-subjects crossover study of E2 effects on the neural processing of threat cues (Fig. 1). In one study arm, participants completed the study visits during the early luteal phase of the menstrual cycle, when endogenous E2 concentrations steeply decline following ovulation. In the other study arm, participants completed the study visits during the early follicular phase of the menstrual cycle, when endogenous E2 concentrations are consistently low.

Prior to menstrual cycle tracking and randomization, we collected self-reports of trauma exposure, and a clinical interview for DSM-5 disorders, including PTSD diagnosis. Participants then began at-home menstrual cycle tracking. They made daily entries in a menstrual cycle-tracking smartphone app, Clue (BioWink GmbH), or via daily text message with study staff, to confirm the onset and duration of menstrual periods. Ovulation timing was determined using urine tests for luteinizing hormone (LH), and participants sent daily photos of each ovulation test to the study team. Participants tracked their cycle for one full baseline month and then were scheduled for MRI visits in the early luteal phase of their cycle (luteal arm) or the early follicular phase (follicular arm). In the luteal arm, when participants recorded a positive LH test, they were contacted to schedule their MRI visits 3 to 7 d later, *M*(*SD*) = 5.5(1.4) days. Because the LH peak occurs 24 to 48 h prior to ovulation, the MRI scans occurred approximately *M* = 3.5 d following ovulation. In the follicular arm, when participants recorded the first day of their menstrual period, they were contacted to schedule their MRI visit within the first 2 to 7 d of the cycle, *M*(*SD*) = 4.5(1.5) days.

Randomization was conducted by a clinical trial coordinator not involved in any patient-facing or data-analytic capacity, using a user-restricted Redcap function (not available to study team members) to begin with either the E2 or placebo condition. The next month, using the same cycle timing procedures (either early luteal or early follicular), participants crossed over and completed the same experimental protocol for the other treatment condition.

Twenty-four hours prior to each MRI scan day, participants came to the laboratory to receive either the E2 or placebo patch. A trained research assistant applied the patch masked with an opaque waterproof bandage to mask any differences between the E2 versus placebo patches, and ensure that the patch would stay in contact with the skin during showering or exercise. Twelve to 24 h after patch administration [when absorption of E2 in circulation has been shown to be highest (55)], participants returned for the MRI scan visit. Following MRI, participants gave a blood sample for assessment of serum hormone levels. Researchers removed and disposed of the patch at the end of each MRI scan visit.

### Estradiol Dose.

E2 patches at a dose of 100ug (Minivelle™) were used. This dose produces an average increase in blood plasma concentration of 65 to 120 pg/mL (56, 57), selected to increase E2 to a level within the normal physiological range for premenopausal women. Average E2 in premenopausal women is <50 pg/mL during the early follicular phase, 300 pg/mL during the late follicular phase, and 190 pg/mL during the luteal phase (56). Shortly after ovulation, E2 levels dip to around 100 pg/mL in the early luteal phase (58).

### Fearful Faces Task.

The fMRI task was selected based on our previous work with trauma-exposed women (10, 59) and men (13). Participants viewed 15 blocks of fearful face stimuli and 15 blocks of neutral face stimuli, with emotion condition pseudorandomly interleaved. Full neuroimaging acquisition details are reported in *SI Appendix*, *Supplemental Methods*.

### Neuroimaging Data Preprocessing and Modeling.

Preprocessing was performed using fMRIPrep 20.2.3 (*SI Appendix*, *Supplemental Methods*). Neural processing of social threat cues was derived from the comparison of fearful > neutral faces. Fearful and neutral face conditions were modeled in SPM12 using a blocked design, convolving the hemodynamic response function with 8-s blocks. First-level models included 6 rigid-body motion parameters, as well as covariates for CSF and WM. Contrast estimates for the fearful > neutral face comparison were extracted from the mean of voxels within anatomically defined deterministic ROIs for the CeA and BLA from the CalTech amygdala atlas (60), as well as the probabilistic ROI for the ComA, thresholded at 75% probability. An ROI for the vmPFC using the boundaries of Brodmann’s area 25 was extracted in the Automated Anatomical Labeling (AAL) software (61). These were exported to R for further analysis.

To investigate regions outside the ROIs, whole-brain exploratory models were conducted using the Sandwich Estimator Toolbox for SPM12 (SwE v.2.2; additional details in *SI Appendix*, *Supplemental Methods*) (62). This toolbox allows for voxel-wise modeling of nested terms, ideal for longitudinal or repeated measures fMRI.

### Serum Hormone Analysis.

After coagulation, samples were spun and aliquoted and frozen at −80°. Serum samples were available for hormone assay from a subset of *n* = 84, across 132 visits. Hormone assays were conducted by the Biomarkers Core Laboratory at the Emory National Primate Research Center. Serum concentrations of 17β-estradiol (E2) and estrone (E1) were measured as a multiplex panel by liquid chromatography-triple quadrupole tandem mass spectrometry (LC–MS/MS; *SI Appendix*, *Supplemental Methods*). Progesterone and testosterone were measured using a multiplex panel by LC–MS/MS. All samples were processed in duplicate, with a mean coefficient of variation of 3.63% for E2, 5.95% for E1, 5.81% for progesterone, and 3.77% for testosterone. Values outside the expected range were verified with a third assay.

### Statistical Analysis.

The primary statistical tests were run in R version 4.3.0, using linear mixed effects models in the lme4 package. Distributions for continuous variables were plotted, assessed for normality, and outliers were winsorized at ±3 SD. Cohen’s d was reported as the effect size estimate for factor variables (E2 vs PB, cycle phase) and R^2^ for dimensional variables. Primary models included the full sample across both the early luteal study arm and early follicular study arm with a term for phase (luteal, follicular), and planned disaggregated models separating each phase were conducted in follow-up analyses. Details of the analysis to address each hypothesis are in *SI Appendix*, *Supplemental Methods*.

## Supplementary Material

Appendix 01 (PDF)

## Data Availability

Data and/or research tools used in the preparation of this manuscript are published in the NDA. Data included in this study are published at http://dx.doi.org/10.15154/93v6-t707 (37). The NDA is a collaborative informatics system created by the NIH to provide a national resource to support and accelerate research in mental health. This manuscript reflects the views of the authors and may not reflect the opinions or views of the NIH.
