# Supplementary material for: Hormonal mechanisms of women’s risk in the face of traumatic stress
Source: Proc Natl Acad Sci U S A. 2025 Dec 15;122(51):e2524903122. doi: 10.1073/pnas.2524903122 (PMC12745815; doi:10.1073/pnas.2524903122)
Supplement: Supplementary file 1 — Appendix 01 (PDF) [file pnas.2524903122.sapp.pdf]

Supplementary Materials

Supplementary Methods
Supplementary Results
Tables S1 to S2
Figs. S1 to S2
References (*1–16*)

Supplementary Methods

Psychological assessment

Assessments of trauma history and psychiatric symptoms were administered at the first laboratory visit, prior to full enrollment into the E2 trial. Diagnostic interviews were conducted by a clinical psychologist or a PhD student supervised by a clinical psychologist. Trauma exposure and current PTSD were assessed using the Clinician Administered PTSD Scale for DSM-5 (1). Diagnoses of current major depressive episode, current alcohol or substance use disorder, and past PTSD using the Diagnostic Interview for Anxiety, Mood, and OCD and Related Neuropsychiatric Disorders (2), or the Mini International Neuropsychiatric Interview version 7.0 (3). These diagnostic interviews also assessed and ruled out psychosis symptoms, for the exclusion criterion. Participants also reported on exposure to childhood adversity using the Childhood Trauma Questionnaire (4), and number and type of lifetime trauma exposures using either the Traumatic Events Inventory (5), or the Life Events Checklist (6). Dissociative symptoms were assessed using the Multiscale Dissociation Inventory (7). On the day of each MRI scan, following the scanning session, participants provided a self-report of current symptoms of PTSD using the PTSD Checklist for DSM-5 (PCL-5) (8), depression using the Beck Depression Inventory (BDI) (9), and anxiety assessed using the State Trait Anxiety Inventory (10).

Serum hormone analysis

Serum samples were available for hormone assay from a subset of n=84, across 132 visits. Hormone assays were conducted by the Biomarkers Core Laboratory at the Emory National Primate Research Center. Serum concentrations of 17β-estradiol (E2) and estrone (E1) were measured as a multiplex panel by liquid chromatography-triple quadrupole tandem mass spectrometry (LC-MS/MS). Serum samples were also analyzed for progesterone and testosterone as a multiplex panel by LC-MS/MS. All samples were processed in duplicate, with a mean coefficient of variation of 3.63% for E2, 5.95% for E1, 5.81% for progesterone, and 3.77% for testosterone. Values outside the expected range were verified with a third assay.

LC-MS/MS protocol for the estrogen multiplex assay. Prior to LC-MS/MS analysis, protein precipitation (PPT) extraction was performed by adding acetonitrile to the subject serum samples. The supernatant, containing the extracted E1-E2, was then separated from the precipitated proteins. Standard, quality control, subject serum samples (250µl each) and 0.75 ml acetonitrile were placed into microcentrifuge tubes, along with 10 µl of internal standards (IS) solution containing final concentrations of 200 pg/ml 17β-Estradiol-d4 (Toronto Research Chemicals, category no. TRC-E888004). After mixing, the tubes were centrifuged at 13,000 rpm for 10 minutes at 4 °C. The supernatant was transferred to a glass culture tube and evaporated to dryness at 42 °C in a Biotage® TurboVap LV evaporation system (Biotage, Uppsala, Sweden). Subsequently, derivatization of E1-E2 was performed by adding 60ul of sodium bicarbonate buffer (50 mM, pH 10.5) into each tube and briefly vortexed, then adding 40 ul of 1mg/ml 1,2-Dimethyl-1H-imidazole-5-sulfonyl chloride in acetone. The samples were vortexed, transferred to microcentrifuge tubes, and incubated for 15 minutes at 60 °C. They were then cooled for 15 minutes at 4 °C. The tubes were then centrifuged at 13,000 rpm for 5 minutes at 4 °C and the supernatant was dried under forced nitrogen at 42 °C in a Biotage® TurboVap LV evaporation system. After reconstitution in 200 µl of 15:85 (v/v) methanol: 0.1 mM ammonium fluoride in water solution containing 0.2% formic acid, samples were analyzed by LC-MS/MS.

The calibration working solution was prepared by dissolving unlabeled E1 (Millipore Sigma, catalog no. E9750) and E2 (Millipore Sigma, catalog no. E8875) in methanol. The calibration standards were made by serially diluting the calibration working solution to yield calibration concentrations ranging from 2 pg/ml – 500 pg/ml for E1 and 1 pg/ml – 500 pg/ml for E2. Calibration samples were prepared by spiking 10 μL of calibration standards and 10 μL of IS, 17β-Estradiol-d4 (IS for E1 and E2) into double charcoal-stripped human serum (BioIVT, NY) to obtain an 8-point calibration curve for each hormone, with coefficients of determination being 0.99 (r2). Quality control samples for E1-E2 were prepared by spiking a pool of human serum with unlabeled solution of each hormone at three different levels: low level (5 pg/ml for E1 and 3.48 pg/ml for E2), medium level (100 pg/ml for E1 and 100.48 pg/ml for E2) and high level (500 pg/ml for E1 and 500.48 pg/ml for E2), and internal standards. Blank samples containing no isotope-labeled standard or unlabeled standards in charcoal-striped serum were also prepared and analyzed.

LC-MS/MS analyses were performed via reverse-phase liquid chromatography using a Shimadzu Nexera 40 Series UHPLC system coupled with a Shimadzu 8060NX triple quadrupole mass spectrometer. Each standard, quality control, or study sample were injected onto a 2.1 mm × 50 mm, 1.7 µm particle Waters Acquity UPLC BEH C18 column with mobile phases composed of 0.1 mM ammonium fluoride in water (A) and methanol (B). The autosampler temperature was 4 °C, and the injection volume was 45 µl A time gradient was created using two LC-40D pumps running in series at a flow rate of 0.4 ml/min. The following gradient elution program was utilized for chromatographic separation: 0.00–1.60 min (10% B), 1.60–4.00 min (10–42% B), 4.00–7.00 min (42–53% B), 7.00–8.50 min (53% B), 8.50–9.40 min (53–60% B), 9.40–11.90 min (60–98% B), 11.90–13.40 min (98% B), 13.40–13.50 min (98%–10% B) and a 1.5 min post-elution period for re-equilibration.

The mass spectrometry system was operated in positive electrospray ionization and multiple reaction monitoring mode. All data were acquired and processed using LabSolutions software 5.118 (Shimadzu, Kyoto, Japan). E1 and E2 concentrations were determined from the standard curve using linear regression analysis. Mean intra- and inter-assay precisions for all quality control (QC) levels were < 9.0 %. Accuracies for standard samples ranged from 85.0% to 114.6%.

LC-MS/MS protocol for the progesterone and testosterone multiplex assay. Before LC-MS/MS analysis, supported liquid extraction (SLE) was employed to extract analytes from 100 µl of each subject's serum. Standard, quality control, and subject serum samples (100µl each) were placed into 96-well microtiter plates, along with 10 µl of internal standards solution containing final concentrations of 10 ng/ml of d3 Testosterone, 10 ng/ml of d9 Progesterone, and 100µl of LCMS grade water. After mixing, the content of each well was transferred to an ISOLUTE® 200 µl Supported Liquid Extraction (SLE) plate and allowed to absorb for 5 min. Analytes were eluted into a 2 ml polypropylene 96-well collection plate (Analytical Sales and Services, Flanders, NJ) via gravity with 1 ml of 50:50 (v/v) dichloromethane/ethyl acetate elution solution. After 5 min, the elution was then completed by applying positive pressure (2.5-5 psi) for 20 seconds. Samples were dried under forced nitrogen at 35 °C in a Biotage® TurboVap 96 automated evaporation system (Biotage, Uppsala, Sweden). After reconstitution in 100 µl of methanol: water (40:60, v/v) solution, samples were analyzed by LC-MS/MS.

The calibration working solution was prepared using unlabeled testosterone solution (1.0 mg/ml testosterone in acetonitrile, Millipore Sigma, category no. T-037), and progesterone solution (1.0 mg/ml progesterone in acetonitrile, Millipore Sigma, category no. P-069). The

calibration standards were made by serially diluting the calibration working solution to yield calibration concentrations ranging from 0.1 ng/ml - 100 ng/ml for testosterone and 0.1 ng/ml - 100 ng/ml for progesterone. Calibration samples were prepared by spiking 10 µL calibration standards and 10 µL of internal standards (d3-testosterone and d9 Progesterone) into double charcoal-stripped human serum (BioIVT, NY) to obtain a 10-point calibration curve for each hormone, with coefficients of determination being 0.999 (r2). Quality control samples for analytes were prepared by spiking a pool of human serum with an unlabeled solution of each hormone at three concentration levels: low level (0.3 ng/ml for testosterone and progesterone), medium level (50 ng/ml for testosterone and progesterone), and high level (100 ng/ml for testosterone and progesterone) and internal standards. Blank samples containing no isotope-labeled standard or unlabeled standards in charcoal-striped serum were also prepared and analyzed.

LC-MS/MS analyses were performed via reverse-phase liquid chromatography using a Shimadzu Nexera X2 UHPLC system coupled with an AB Sciex Triple Quad 6500 Mass Spectrometer. Each standard, quality control, or study sample were injected onto a 4.6mm×50 mm, 3.5 µm particle Agilent C18 XDB column with mobile phases composed of water containing 0.1% (v/v) formic acid and 5mM Ammonium acetate (A) and methanol containing 0.1% (v/v) formic acid and 5mM Ammonium acetate (B). The autosampler temperature was 4 °C and the injection volume was 15 µl. A time gradient was created using 2 LC-30AD pumps running in series at a flow rate of 0.5 ml/min. The following gradient elution program was utilized for chromatographic separation: 0–0.01 min (40% B), 0.01–1.2 min (40–95% B), 1.2–4.2 min (95% B), 4.2–4.25 min (95–40% B), and a 1.25 min post-elution period for re-equilibration.

The mass spectrometry system was operated in positive electrospray ionization and multiple reaction monitoring mode. All data were acquired and processed using Analyst® Software 1.7. Samples were assayed in duplicates, and hormone levels were determined from the standard curve using linear regression analysis. Mean intra- and inter-assay precisions for all quality control (QC) levels were < 12.6%. Accuracies for standard samples ranged from 86.0% to 115.0%.

Neuroimaging data acquisition

Scanning took place on a 3T Siemens Trio (Siemens, Malvern, PA), using a 32-channel head coil. Mid-way through the study, the neuroimaging center elected to upgrade the scanner to a Prisma-Fit model, resulting in n=40 scanned on the Trio and n=70 scanned on the Prisma. We used the same sequences and parameters for the acquisition protocol on both models, and conducted pilot testing to ensure similar signal to noise ratio (SNR) across the two acquisition protocols. A T1w MPRAGE was collected for the assessment of gray matter anatomy (TR=2400ms, TE=2.07ms, FA=8°, 0.8mm^3 voxel size). After data quality assessment mid-way through the study, the “prescan normalize” option was activated to enhance signal homogeneity during acquisition of the T1w image; this was tagged as a covariate in all neuroimaging models. Parallel imaging (GRAPPA) with an acceleration factor of 2 was used. For fMRI scans, multi-echo multiband T2* echo-planar images were collected with 48 slices in an ascending interleaved sequence (TR=1100ms, TE 1/2/3=15.00/37.14/59.28, 2.5mm^3 voxel size, phase encoding P>>A, field of view = 210 x 210 x 120mm), and multiband acceleration factor of 4. Parallel imaging (GRAPPA) with an acceleration factor of 2 was used.

Fearful faces fMRI task: Participants viewed 15 blocks of fearful face stimuli and 15 blocks of neutral face stimuli, with emotion condition pseudo-randomly interleaved. Within each block, eight face stimuli were presented, with the face presented for 500ms, followed by a 500ms ITI. After every tenth block, participants were instructed to rest and relax for 10,000ms. Participants were engaged in passive viewing of the faces, to minimize any influence of cognitive task on the emotional arousal response to the face stimuli.

Neuroimaging data preprocessing

Results included in this manuscript come from preprocessing performed using fMRIPrep 20.2.3 (RRID:SCR_016216), which is based on Nipype 1.6.1 (RRID:SCR_002502).

Anatomical data preprocessing. A total of 1 T1-weighted (T1w) images were found within the input BIDS dataset. The T1-weighted (T1w) image was corrected for intensity non-uniformity (INU) with N4BiasFieldCorrection (11), distributed with ANTs 2.3.3 (RRID:SCR_004757), and used as T1w-reference throughout the workflow. The T1w-reference was then skull-stripped with a Nipype implementation of the antsBrainExtraction.sh workflow (from ANTs), using OASIS30ANTs as target template. Brain tissue segmentation of cerebrospinal fluid (CSF), white-matter (WM) and gray-matter (GM) was performed on the brain-extracted T1w using fast (FSL 5.0.9, RRID:SCR_002823). Brain surfaces were reconstructed using recon-all (FreeSurfer 6.0.1, RRID:SCR_001847), and the brain mask estimated previously was refined with a custom variation of the method to reconcile ANTs-derived and FreeSurfer-derived segmentations of the cortical gray-matter of Mindboggle (RRID:SCR_002438). Volume-based spatial normalization to one standard space (MNI152NLin2009cAsym) was performed through nonlinear registration with antsRegistration (ANTs 2.3.3), using brain-extracted versions of both T1w reference and the T1w template. The following template was selected for spatial normalization: ICBM 152 Nonlinear Asymmetrical template version 2009c [RRID:SCR_008796; TemplateFlow ID: MNI152NLin2009cAsym],

Functional data preprocessing. First, a reference volume and its skull-stripped version were generated from the shortest echo of the BOLD run using a custom methodology of fMRIPrep. Susceptibility distortion correction (SDC) was omitted. The BOLD reference was then co-registered to the T1w reference using bbregister (FreeSurfer) which implements boundary-based registration (12). Co-registration was configured with six degrees of freedom. Head-motion parameters with respect to the BOLD reference (transformation matrices, and six corresponding rotation and translation parameters) are estimated before any spatiotemporal filtering using mcflirt (FSL 5.0.9) (13). BOLD runs were slice-time corrected using 3dTshift from AFNI 20160207 (RRID:SCR_005927). The BOLD time-series (including slice-timing correction when applied) were resampled onto their original, native space by applying the transforms to correct for head-motion. These resampled BOLD time-series will be referred to as preprocessed BOLD in original space, or just preprocessed BOLD. A T2* map was estimated from the preprocessed BOLD by fitting to a monoexponential signal decay model with nonlinear regression, using T2*/S0 estimates from a log-linear regression fit as initial values. For each voxel, the maximal number of echoes with reliable signal in that voxel were used to fit the model. The calculated T2* map was then used to optimally combine preprocessed BOLD across echoes following the method described in (14). The optimally combined time series was carried forward as the preprocessed BOLD. First, a reference volume and its skull-stripped version were generated using a custom methodology of fMRIPrep. The BOLD time-series were resampled into standard space, generating a preprocessed BOLD run in MNI152NLin2009cAsym space.

Several confounding time-series were calculated based on the preprocessed BOLD: framewise displacement (FD), DVARS and three region-wise global signals. FD was computed using two formulations following Power (absolute sum of relative motions, (15)) and Jenkinson (relative root mean square displacement between affines, (13)). FD and DVARS are calculated for each functional run, both using their implementations in Nipype (following the definitions by (15)). The three global signals are extracted within the CSF, the WM, and the whole-brain masks. The head-motion estimates calculated in the correction step were also placed within the corresponding confounds file. The confound time series derived from head motion estimates and global signals were expanded with the inclusion of temporal derivatives and quadratic terms for each (16). All resamplings can be performed with a single interpolation step by composing all the pertinent transformations (i.e. head-motion transform matrices, susceptibility distortion correction when available, and co-registrations to anatomical and output spaces). Gridded (volumetric) resamplings were performed using antsApplyTransforms (ANTs), configured with Lanczos interpolation to minimize the smoothing effects of other kernels. Many internal operations of fMRIPrep use Nilearn 0.6.2 (RRID:SCR_001362), mostly within the functional processing workflow. For more details of the pipeline, see the section corresponding to workflows in fMRIPrep's documentation. Following preprocessing, the images were smoothed using a 6mm Gaussian kernal using the 3dBlurToFWHM routine in AFNI (RRID:SCR_005927).

Statistical analysis for hypothesis testing

We first conducted a manipulation check to assess whether the E2 patch produced an increase in circulating estradiol levels by the time of the scan, modeling estradiol (pg/mL) as a function of patch condition (E2, placebo), menstrual cycle phase (early follicular, early luteal), and their interaction, with a covariate for visit (visit 1, visit 2), and random effects term for participant.

To test the hypothesis that E2 administration would reduce amygdala responses and increase vmPFC responses to social threat cues, the fearful>neutral contrast estimate within each ROI was modeled as a function of patch condition (E2, PB), cycle phase (early follicular, early luteal), and their interaction, with covariates for visit (visit 1, visit 2), and prescan normalize, and a random effects term for participant. This produced 7 models (right CeA, left CeA, right ComA, left ComA, right BLA, left BLA, and the bilateral vmPFC ROI), and the results were thresholded using a Bonferroni correction for the number of tests resulting in a final $p<0.01$ threshold. Planned follow-up models separately assessed E2 patch effects within each menstrual cycle phase individually.

To test for trauma- or PTSD-related group differences, we first investigated the neural responses to threat cues (Fearful > Neutral contrast) during the placebo condition alone. Linear mixed effects models using *lmer* modeled each ROI as a function of group (PTSD+, TC, NLT), menstrual cycle phase (early follicular, early luteal), and their interaction, with covariates for visit (1, 2), and prescan normalize. TC was used as the reference group, as this group differs from the PTSD group in diagnosis but not trauma exposure history, and differs from the NLT group in trauma exposure history but not diagnosis. Bonferroni correction was applied for the 5 tests (one per ROI).

To test the hypothesis that E2 may have different effects on threat processing in the PTSD+, TC, and NLT groups, each ROI was modeled as a function of patch condition (E2, PB), cycle phase (early follicular, early luteal), group (PTSD+, TC, NLT) and their 2- and 3-way interactions, with covariates for visit (visit 1, visit 2), and prescan normalize, and a random

effects term for participant. TC was again used as the reference group. Bonferroni correction was again applied for the 7 tests (one per ROI).

We also conducted exploratory whole-brain analysis. The whole-brain analysis tested whether exogenous E2 may have trauma- or PTSD-specific effects on regions outside the amygdala and vmPFC ROIs. Therefore, an E2 patch (E2, PB) x group (PTSD+, TC, NLT) x phase (early follicular, early luteal) model was tested, with covariates for visit, and prescan normalize. Participant was modeled as a random effect, and within-subject terms (E2 patch, visit) were nested within participant. A non-parametric wild bootstrap approach was used for thresholding, with 999 bootstraps, and type C2 sample size adjustment, under an unrestricted SwE (U-SwE). An initial cluster-forming threshold was set at $p<0.005$, with extent correction applied to reach a false discovery rate (FDR)-corrected 0.05.

Finally, to examine the effects of E2 administration of self-reported symptoms and sympathetic arousal, each outcome was measured as a function of patch condition (E2, placebo), menstrual cycle phase (early follicular, early luteal), and their interaction, with a covariate for visit (visit 1, visit 2), and random effects term for participant.

Supplementary Results

Effects of E2 on depression symptoms

We also investigated whether the E2 patch had any effect on reported symptoms of depression on the BDI. These were reported for the prior 30 days, with patch days reflecting only one day earlier, and current day of the visit), and we therefore did not expect any effect of the patch. This was the confirmed; there was no effect of E2 administration on depression symptom severity (E2 main effect $p= 0.93$, E2 x groupPTSD+ $p=0.27$).

Table S1. E2 x phase effects on neural response to social threat cues (fearful > neutral faces): vmPFC ROI

	vmPFC		
Predictors	*Estimate*	*SE*	*p*
(Intercept)	0.01	0.01	0.385
patch [E2]	-0.02	0.01	**0.002**
phase [luteal]	-0.00	0.01	0.738
visit	-0.00	0.00	0.686
prescan normalize	0.00	0.00	0.652
patch [E2] × phase [luteal]	0.02	0.01	0.055
**Random Effects**			
σ^2	0.00		
$\tau_{00\ sid}$	0.00		
Observations	203		
Marginal R^2	0.046		

Note. P-values < 0.007 were considered significant after Bonferroni correcting for 7 ROIs, and significant values are highlighted in bold. For categorical variables, the reference categories were PB for the patch term, follicular for the phase term.

Table S2. Group x E2 x phase effects on neural response to social threat cues (fearful > neutral faces): right CeA and right ComA ROIs

	Right CeA			Right ComA		
Predictors	*b*	*SE*	*p*	*b*	*SE*	*p*
(Intercept)	0.06	0.05	0.207	0.13	0.05	0.016
patch [E2]	-0.02	0.04	0.597	-0.09	0.05	0.092
phase [luteal]	-0.15	0.05	**0.002**	-0.15	0.06	0.011
group [NLT]	-0.09	0.05	0.067	-0.06	0.05	0.243
group [PTSD]	-0.05	0.05	0.300	-0.06	0.05	0.269
visit	0.03	0.02	0.089	-0.01	0.02	0.704
prescan normalize	0.01	0.02	0.665	0.02	0.02	0.502
patch [E2] × phase [luteal]	0.16	0.07	0.018	0.24	0.08	**0.003**
patch [E2] × group [NLT]	0.02	0.06	0.737	0.05	0.08	0.489
patch [E2] × group [PTSD]	0.03	0.07	0.654	0.06	0.08	0.426
phase [luteal] × group [NLT]	0.23	0.07	**0.001**	0.23	0.08	**0.004**
phase [luteal] × group [PTSD]	0.15	0.07	0.044	0.14	0.08	0.082
(patch [E2] × phase [luteal]) × group [NLT]	-0.28	0.10	**0.005**	-0.34	0.11	**0.003**
(patch [E2] × phase [luteal]) × group [PTSD]	-0.18	0.10	0.069	-0.17	0.12	0.150
**Random Effects**						
σ^2	0.02			0.03		
$\tau_{00\ subj}$	0.00			0.00		
Observations	203			203		
Marginal R^2	0.09			0.09		

Note. P-values < 0.007 were considered significant after Bonferroni correcting for 7 ROIs, and significant values are highlighted in bold. For categorical variables, the reference categories were PB for the patch term, follicular for the phase term, TC for the group term.

Figure S1.

Follicular study arm

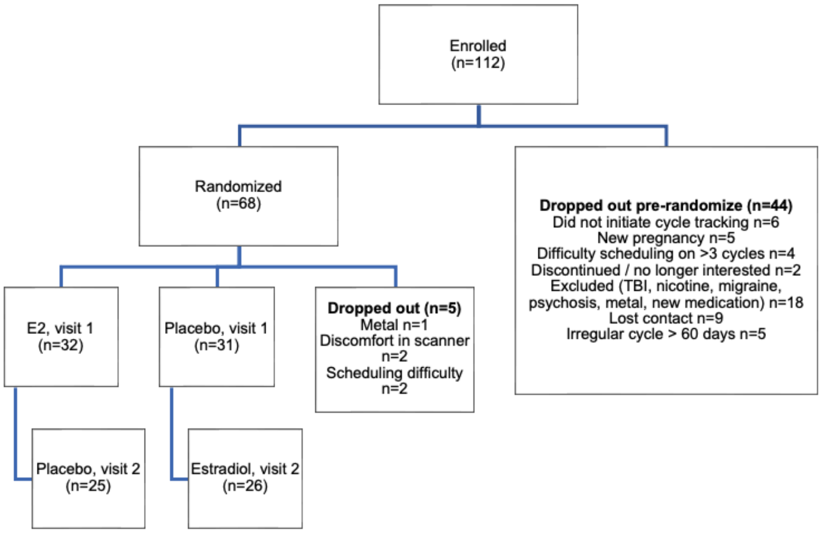

Luteal study arm

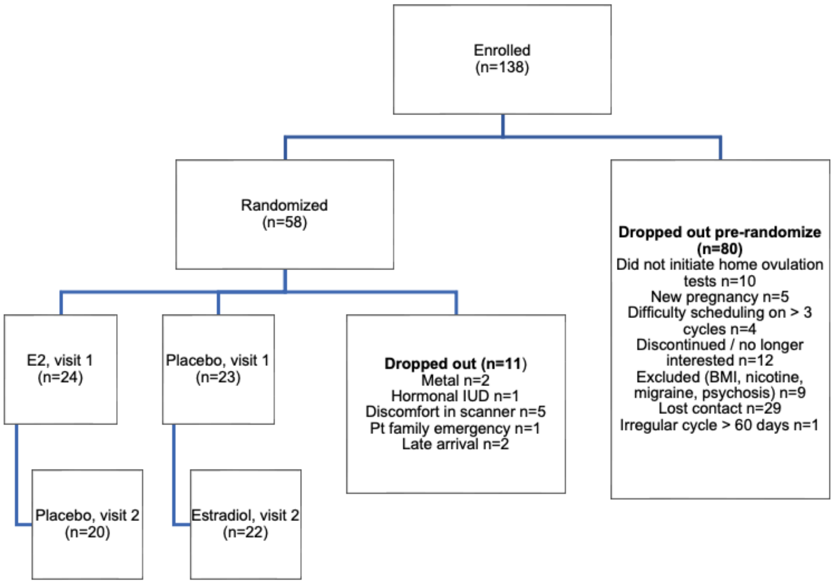

Figure S1. Cohort diagram. Participants in the study were enrolled after an initial psychological assessment visit, and began tracking their menstrual cycles. Participants then completed at least one initial baseline month of cycle tracking. On a subsequent cycle, when they reached either menses +2-7 days (follicular arm of the study), or ovulation +3-7 days (luteal arm of the study), they returned to the lab for randomization to either the E2 or PB patch, and completed an MRI visit. In a subsequent month, at the same time of the cycle, they crossed over to the other study condition (either E2 or placebo) and completed another MRI visit. Dropouts at each stage of the study are shown here, along with final sample included in the analysis.

Figure S2.

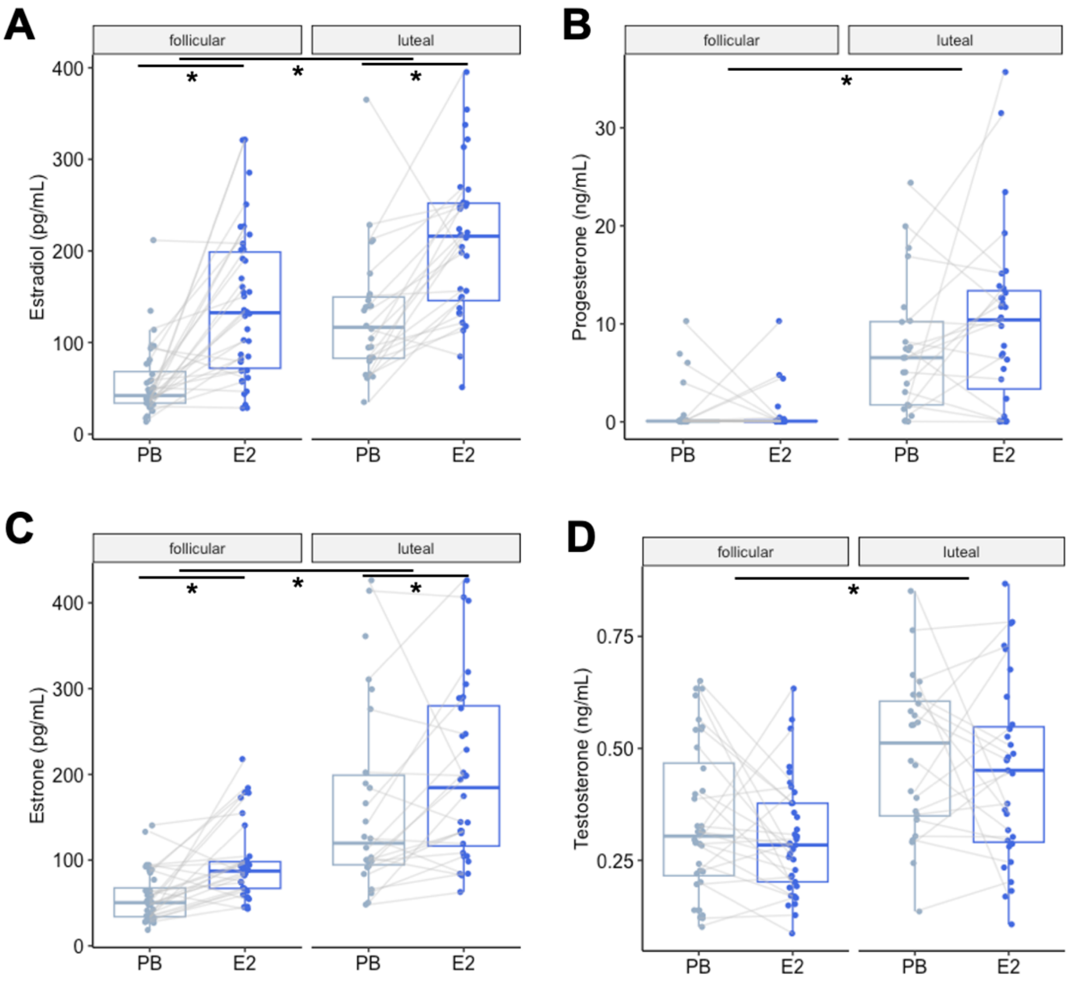

Figure S2. Serum hormone levels as a function of E2 patch condition and menstrual cycle phase. A. Exogenous E2 produced a significant increase in serum estradiol relative to placebo, in both the early follicular phase and the early luteal phase. Described further in Figure 1C. B. Plasma progesterone (P4) levels were not significantly influenced by the administration of exogenous E2 (Main effect of E2 patch p=0.90, E2 * cycle phase interaction p=0.51), but progesterone was higher in the luteal phase compared to the follicular phase (t=4.19, p<0.001). C. Serum estrone levels were increased with the administration of exogenous E2 (Main effect of E2 patch t=3.19, p=0.002, E2 * cycle phase interaction p=0.62), and were higher in the luteal phase compared to the follicular phase (t=6.38, p<0.001). D. Plasma testosterone levels were not significantly influenced by the administration of exogenous E2 (Main effect of E2 patch p=0.16, E2 * cycle phase interaction p=0.92), but testosterone was higher in the luteal phase compared to the follicular phase (t=3.39, p<0.001).

References:

1. F. W. Weathers, *et al.*, The Clinician-Administered PTSD Scale for DSM–5 (CAPS-5): Development and initial psychometric evaluation in military veterans. *Psychological Assessment* **30**, 383–395 (2018).

2. D. F. Tolin, *et al.*, Diagnostic Interview for anxiety, mood, and OCD and related neuropsychiatric disorders (DIAMOND). *Institute of Living/Hartford HealthCare Corporation* 1–128 (2016).

3. D. Sheehan, *et al.*, The MINI international neuropsychiatric interview (Version 7.0. 2) for DSM-5. *Harm Research Institute* (2016).

4. D. P. Bernstein, L. Fink, L. Handelsman, J. Foote, Childhood Trauma Questionnaire. https://doi.org/10.1037/t02080-000. Deposited 12 September 2011.

5. A. C. Schwartz, R. L. Bradley, M. Sexton, A. Sherry, K. J. Ressler, Posttraumatic Stress Disorder Among African Americans in an Inner City Mental Health Clinic. *PS* **56**, 212–215 (2005).

6. M. J. Gray, B. T. Litz, J. L. Hsu, T. W. Lombardo, Psychometric Properties of the Life Events Checklist. *Assessment* **11**, 330–341 (2004).

7. J. Brière, *MDI, Multiscale dissociation inventory: Professional manual* (Psychological Assessment Resources, Incorporated, 2002).

8. C. A. Blevins, F. W. Weathers, M. T. Davis, T. K. Witte, J. L. Domino, The Posttraumatic Stress Disorder Checklist for DSM-5 (PCL-5): Development and Initial Psychometric Evaluation. *Journal of Traumatic Stress* **28**, 489–498 (2015).

9. A. T. Beck, C. H. Ward, M. Mendelson, J. Mock, J. Erbauch, Beck Depression Inventory. https://doi.org/10.1037/t00741-000. Deposited 12 September 2011.

10. C.D. Spielberger, R.L. Gorsuch, R.E. Lushene, P.R. Vagg, G.A. Jacobs, “Manual for the state-trait anxiety inventory (Form Y)” in (Consulting Psychologists Press, 1983).

11. N. J. Tustison, *et al.*, N4ITK: Improved N3 Bias Correction. *IEEE Transactions on Medical Imaging* **29**, 1310–1320 (2010).

12. D. N. Greve, B. Fischl, Accurate and robust brain image alignment using boundary-based registration. *NeuroImage* **48**, 63–72 (2009).

13. M. Jenkinson, P. Bannister, M. Brady, S. Smith, Improved Optimization for the Robust and Accurate Linear Registration and Motion Correction of Brain Images. *NeuroImage* **17**, 825–841 (2002).

14. S. Posse, *et al.*, Enhancement of BOLD-contrast sensitivity by single-shot multi-echo functional MR imaging. *Magnetic Resonance in Medicine* **42**, 87–97 (1999).

15. J. D. Power, *et al.*, Methods to detect, characterize, and remove motion artifact in resting state fMRI. *NeuroImage* **84**, 320–341 (2014).

16. T. D. Satterthwaite, *et al.*, An improved framework for confound regression and filtering for control of motion artifact in the preprocessing of resting-state functional connectivity data. *NeuroImage* **64**, 240–256 (2013).
